# Accuracy of PSI-based hybrid workflow using a temporary intermediate splint versus conventional splint-based maxillary positioning in orthognathic surgery: a retrospective cohort study

**DOI:** 10.1186/s12903-026-09695-4

**Published:** 2026-08-25

**Authors:** Jonathan Bertram, Henry Leonhardt, Fred Podmelle, Thomas Teltzrow, Adrian Franke

**Affiliations:** 1 Department of Oral and Maxillofacial Surgery, Facial Plastic Surgery, Ernst-Von-Bergmann Klinikum Potsdam, Charlottenstraße 72, Potsdam, 14467 Germany; 2 Department of Oral and Maxillofacial Surgery, Medical Faculty and University Hospital Carl Gustav Carus, Technische Universität Dresden, Fetscherstraße 74, Dresden, 01307 Germany; 3 Department of Oral and Maxillofacial Surgery, Medical Faculty and University Hospital Greifswald, Fleischmannstraße 8, Greifswald, 17475 Germany

**Keywords:** Orthognathic surgery, Maxillo-mandibular surgery, CAD-CAM, Mandibular advancement splints, Maxillary osteotomy

## Abstract

**Background:**

This retrospective cohort study, adhering to STROBE guidelines, compared the accuracy of maxillary positioning achieved with a patient-specific implant (PSI)-based hybrid workflow, which utilised a temporary intermediate splint to identify and remove posterior bony interferences before definitive splintless PSI fixation, to conventional splint-based positioning using stock titanium osteosynthesis plates in orthognathic surgery. Translational and rotational movement deviations from the virtual surgical plan were assessed.

**Methods:**

Twenty-six patients who underwent non-segmental Le Fort I osteotomy between January 2020 and June 2023 were included. Eleven patients received the PSI-based hybrid workflow, while 15 patients underwent conventional splint-based positioning. Group allocation was based on the logistical feasibility of PSI planning and manufacturing rather than randomisation. Pre- and postoperative 3D images were superimposed using IPS® Case Designer software (KLS Martin) to quantify deviations in three translational and three rotational axes. Between-group comparisons were conducted using unpaired *t*-tests and two-way ANOVA, and correlations were assessed using Pearson’s coefficient.

**Results:**

The mean translational deviation was significantly lower in the PSI group compared to the control group (0.46 ± 0.33 mm vs 0.79 ± 0.58 mm; *p* = .0044), as was the mean rotational deviation (0.82° ± 0.91° vs 1.24° ± 0.85°; *p* = .0397). Significant differences were identified for vertical translation (*p* = .0162) and yaw rotation (*p* = .0205). Operating time did not differ significantly between groups (*p* = . 1739). In the PSI group, greater planned displacement showed a weak correlation with larger deviations, a relationship not observed in the control group.

**Conclusions:**

The PSI-based hybrid workflow resulted in statistically greater positioning accuracy compared to conventional splint-based fixation. However, the absolute improvements (approximately 0.3 mm and 0.5°) were small, likely of limited clinical relevance, and were not associated with reduced operating times. Due to the retrospective, non-randomised design, small sample size, and use of a PSI-based hybrid protocol, these findings should be considered hypothesis-generating. Larger prospective studies evaluating fully splintless PSI workflows and patient-centred outcomes are recommended.

**Clinical trial number:**

Not applicable.

## Background

Orthognathic surgery constitutes a complex and essential component of oral and maxillofacial surgery. Tooth-supported surgical splints are commonly used to position the upper and lower jaws. However, precise placement of the upper jaw in a predetermined position is susceptible to error, as this process relies on rotation of the maxillomandibular complex within the temporomandibular joint [1, 2], which lacks rotational stability during movement [3]. The literature reports that maxillary deviation from the planned position using splint-based maxillary positioning is approximately 2 mm [4–6]. Patient-specific implants (PSIs) have been introduced to mitigate this error by eliminating the manual, rotation-dependent transfer step at the mandibular joint [7].

The concept of using PSIs to prevent this error has been recognised for several decades. In 1987, osteosynthesis plates were first pre-bent on a 3D-printed model and subsequently used for upper jaw positioning [8], a highly time-consuming process. The introduction of computer-aided design (CAD) for maxillary position planning, combined with laser-sintered titanium implants, enabled the widespread adoption of PSIs in orthognathic surgery from 2013 onward [9].

Subsequently, numerous studies have investigated the potential for improved accuracy with PSIs. Most have examined translational movements as the primary focus [5, 10–13], whereas fewer have addressed rotational movements, which are essential for a comprehensive evaluation of accuracy [14–16]. The literature remains inconsistent: some studies report no significant difference in accuracy between PSI and splint-based techniques [13, 16], while others demonstrate a clear improvement with PSIs [10–12, 16, 17].

This study evaluates the accuracy of a PSI-based hybrid workflow, which combines temporary splint-guided removal of posterior bony interferences with definitive splintless PSI fixation, compared with conventional splint-based maxillary positioning. The analysis examines both translational and rotational movements and hypothesises that the PSI-based hybrid workflow achieves greater precision.

## Methods

### Study design, setting, and participants

A retrospective single-centre cohort study was conducted according to the STROBE guidelines, including patients who underwent orthognathic surgery between January 2020 and June 2023 in the Department of Oral and Maxillofacial Surgery and Facial Plastic Surgery at Ernst von Bergmann Hospital, Potsdam, Germany. The study was carried out in accordance with the guiding principles of the Declaration of Helsinki, and the Ethics Committee of the State Medical Association of Brandenburg, Germany, granted ethical approval (Ref. No. 2023–96-BO-ff).

Patients were allocated to one of two non-randomised groups: an interventional cohort treated with cutting guides and 3D-laser-sintered titanium PSIs using a hybrid, temporary-splint-assisted workflow (PSI Group), and a control group treated with conventional splint-based maxillary positioning and fixation using stock titanium implants (control group).

The inclusion criteria comprised non-segmental Le Fort I osteotomy, absence of intraoperative changes to the planned upper jaw position, maxilla-first surgery, a standardised operative procedure, no significant intraoperative complications related to positioning, and performance by the same surgical team. Only primary maxillary or bimaxillary osteotomies were considered. Exclusion criteria included syndromic disease, a history of or current cleft lip and palate, mandibular-first surgery, or secondary orthognathic surgery.

Patients were not randomised to treatment groups. Allocation to the PSI group occurred between November 2021 and June 2023. Group assignment was primarily determined by the availability of sufficient preoperative time for virtual planning, manufacturing, and sterilisation of the PSI before surgery. Patients for whom this logistical timeframe could not be met underwent conventional splint-based surgery and were assigned to the control group. As allocation was based on logistical rather than random criteria, systematic differences between groups related to case timing or complexity may be present.

### Variables

Patient demographic data, including age, gender, and Angle classification, were collected. The primary outcome measure was the difference between the virtually planned and the final position of the maxilla. Translational (vertical, horizontal, sagittal) and rotational (roll, pitch, yaw) deviations from the planned position were measured along all three axes and reported in millimetres (mm) and degrees (°), and reported as overall absolute translational and rotational deviations, respectively. The secondary outcome measure were absolute deviations for each of the three translational and rotational axes, operating time, defined as the interval from the initial incision to the completion of the surgical suture, as documented in the surgical report, and the relationship between planned movement magnitude and achieved deviation.

The exposure under investigation was the PSI-based hybrid workflow, which was compared to the splint-based technique. No predictors for outcome were included. Age, Angle classification, surgeon experience, and rater reliability were considered potential confounders and were addressed accordingly. Effect modifiers were controlled through the inclusion and exclusion criteria.

### Procedures

#### Virtual surgical planning and splint manufacturing (both groups)

**Fig. 1:**
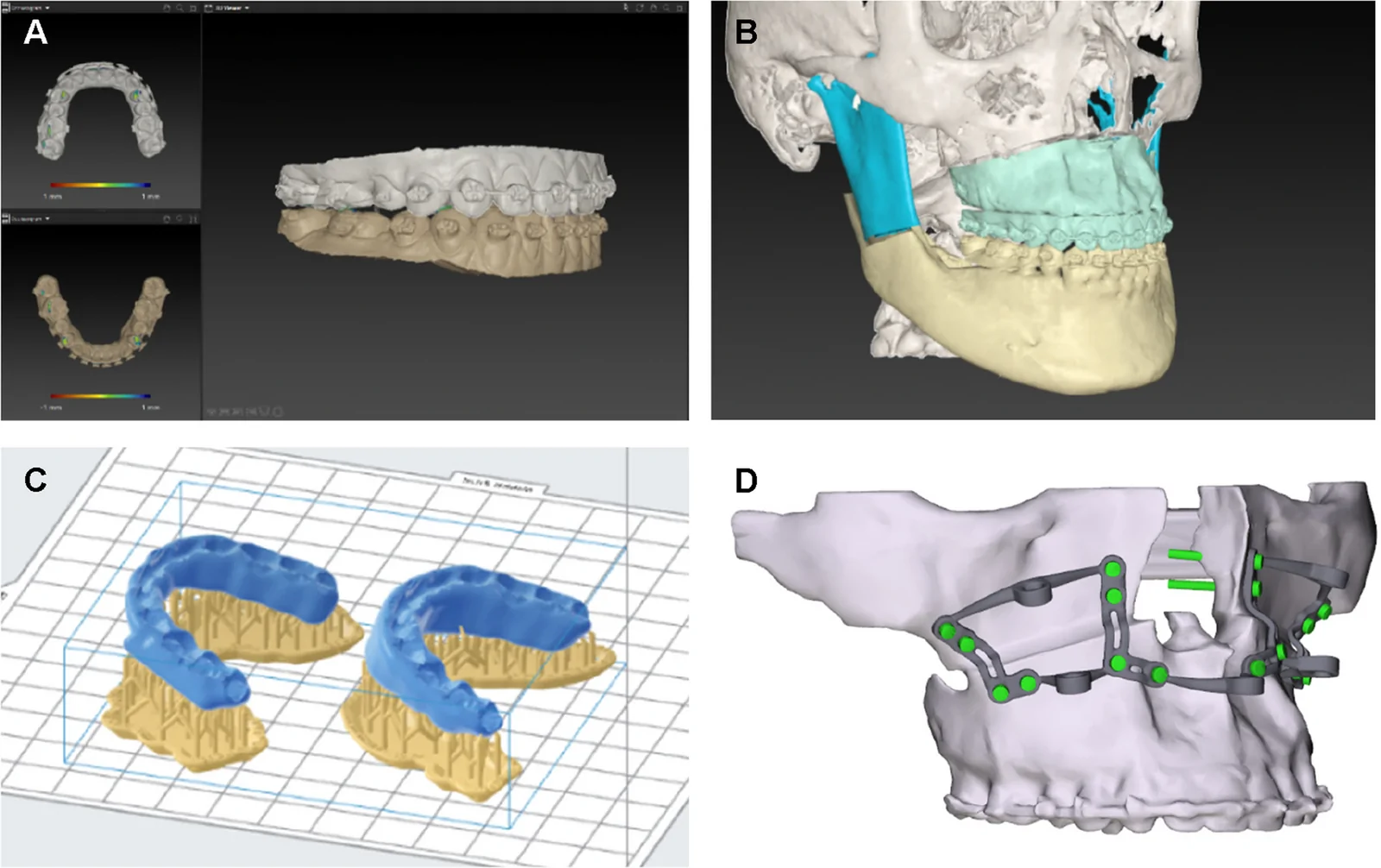
Depicts the sequential stages involved in creating surgical splints and PSIs for orthognathic surgery. **A** Virtual occlusion is established using the IPS® Case Designer from KLS Martin. **B** Bimaxillary surgery is virtually planned with the same software. **C** Surgical splints are prepared for 3D using PreForm® software. **D** The virtual planning of the PSI is completed prior to surgical approval, with KLS Martin responsible for the PSI planning

Osteotomies for both groups were planned using the IPS® Case Designer (Version 2.7.5.1, KLS Martin, Tuttlingen, Germany). DICOM datasets and intraoral scanner STL files were imported into the software. Habitual occlusion was disoriented by a light bite on cotton rolls following masticatory muscle massage. Centric occlusion was then located in the supine position and fixed with a wax bite plate. The planning cone beam computed tomography (CBCT) was acquired in this centric bite fixation. The DICOM datasets were obtained from either a KaVo 3D eXam device (23 × 17 cm, voxel size 0.3 mm) or a Planmeca Viso® G5 (20 × 17 cm, voxel size 0.3 mm), depending on the consultation office. The 3D surface calculated by the software was matched with the STL data from an intraoral scanner (CareStream CS 3600). The target occlusion was determined using the “Virtual Occlusion” tool of IPS® Case Designer (Fig. 1A). Osteotomy lines in the maxilla and mandible were set according to the target occlusion, and translational and rotational movements were defined (Fig. 1B). Soft-tissue prediction was generated, and the planned movements and predicted appearance were discussed with the patient.

Intermediate and final splints were designed as STL datasets that captured all planned movements of the mandible and maxilla. These datasets were imported into PreForm® software (Version 3.43.1.462, Formlabs Inc., Somerville, MA, USA) and printed on a Form 2 3D printer (Formlabs Inc., Somerville, MA, USA) using Formlabs Surgical Guide Resin (RS-F2-SGAM-01) (Fig. 1C). Printed splints were washed in 95% isopropyl alcohol for 20 min, cured at 60 °C for 60 min in a light-curing oven, support structures were removed, and the splints were steam-sterilised at 121 °C for 15 min. For the PSI group, the surgical plan was also transmitted to KLS Martin for manufacture of the laser-sintered titanium implant via IPS® Gate (Fig. 1D).

#### Intervention group (PSI group): PSI-based hybrid maxillary positioning

**Fig. 2:**
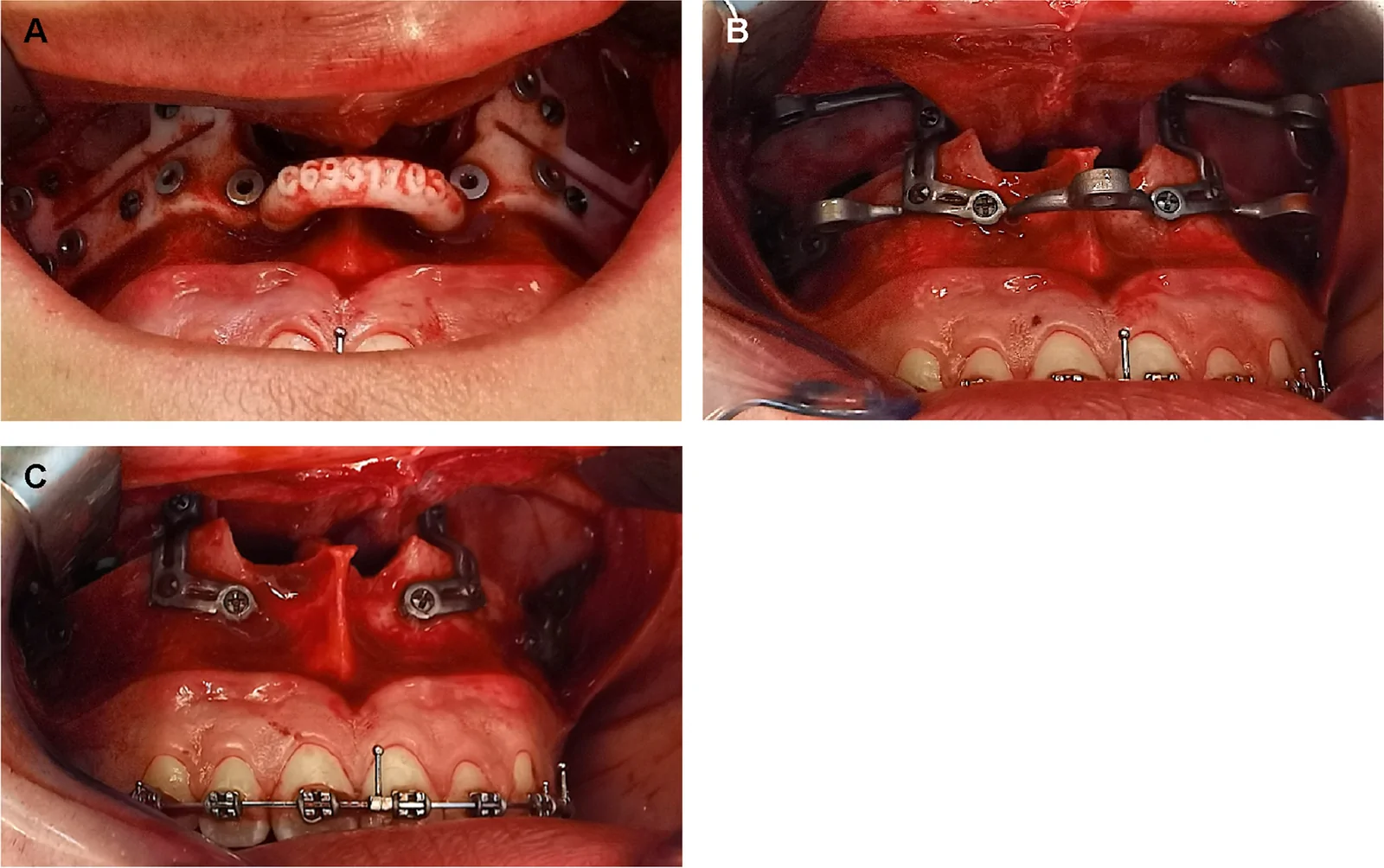
Illustrates the use of a drilling template and a PSI from KLS Martin to achieve maxillary displacement. **A** The KLS Martin drilling template is inserted with three fixation screws following visualisation of the bony maxilla. **B** The maxilla is displaced in the Le Fort I plane, and the KLS Martin PSI is used for osteosynthesis. The implant stabilisation bridges are still in place. **C** The final situation after osteosynthesis and removal of the implant stabilisation bridges of the PSI

Within the PSI group, Le Fort I osteotomy was conducted using a combined cutting and drill guide (Fig. 2A), which was secured with seven 6 mm screws. Drill holes were then created, and the cutting lines were osteotomised. Following removal of the cutting guide, the osteotomy line was completed, and the down-fracture was performed.

**Fig. 3:**
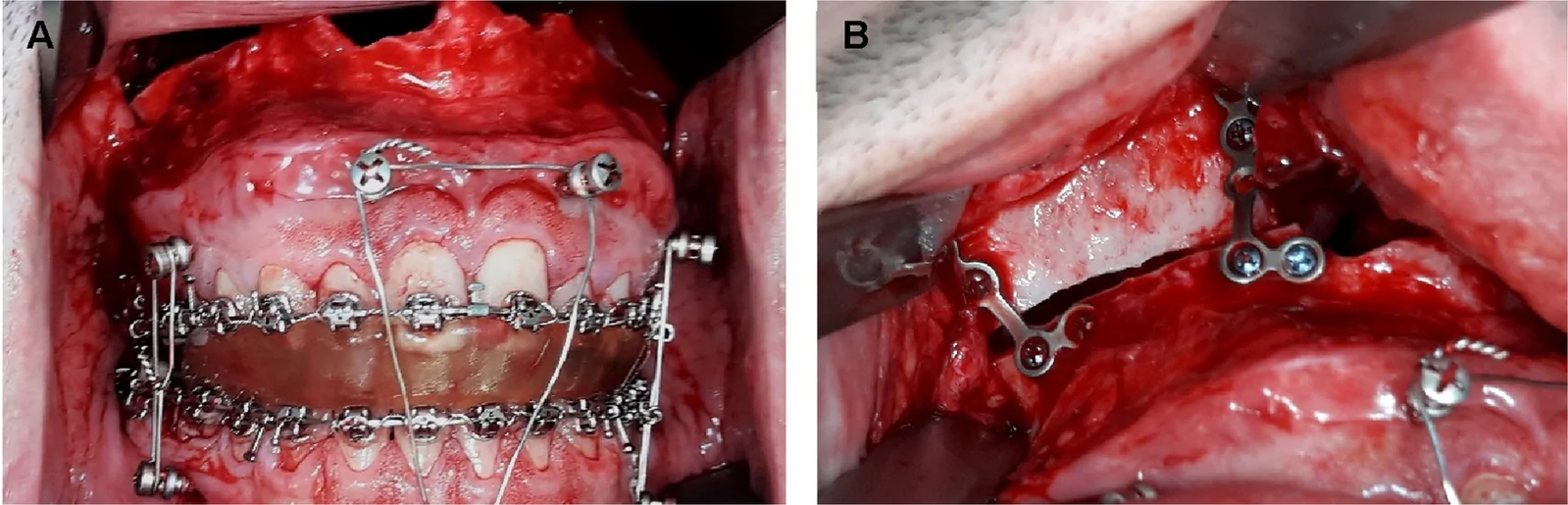
Shows maxillary displacement achieved with a surgical splint and the fixation of the maxilla using stock osteosynthesis plates. **A** The intermediate splint is fixed with maxillomandibular wire fixation. **B** Two 5-hole L-osteosynthesis plates from KLS Martin on the right maxilla

To eliminate bone interferences in the distal maxilla, an intermediate splint was inserted and secured with wires between the jaws (Fig. 3A) [12, 18]. This splint was used solely to identify and remove posterior bony interferences and was not intended to determine the final maxillary position. After removal of the wire fixation and intermediate splint, the PSI was inserted, without a splint, into the pre-drilled positions and fixed with 5 mm screws (Fig. 2B). The implant stabilisation bridges were then removed, leaving four L-shaped plates with four screws each (REF 25–875-05–09, KLS Martin SE & Co. KG) to fix the maxilla (Fig. 2C).

#### Comparator (Control group): conventional splint-based maxillary positioning

In the control group, the Le-Fort I osteotomy was conducted according to the established surgical technique [1, 18]. The upper jaw was positioned with an intermediate splint and secured between the maxilla and mandible using at least six MMF screws (REF 25–092-08–09 and REF 25–092-12–09, KLS Martin SE & Co. KG, KLS Martin Platz 1, 78,532 Tuttlingen, Germany) (Fig. 3A). The target position of the upper jaw was determined by rotating the maxillomandibular complex at the temporomandibular joint. Maxillary osteosynthesis was completed using four L-shaped plates (REF 50–381-05–09, KLS Martin SE & Co. KG, KLS Martin Platz 1, 78,532 Tuttlingen, Germany) (Fig. 3B).

#### Bimaxillary procedures and postoperative protocol

For patients undergoing for bimaxillary surgery, both groups received a conventional sagittal-split osteotomy. In the control group, vertical (cranio-caudal) positioning of the maxilla was assessed intraoperatively using paranasal reference drillings. Two reference holes were placed on each side of the piriform aperture: the superior drill hole at a fixed point on the piriform rim or paranasal bone above the osteotomy line, and the inferior hole 10 mm below the osteotomy line. The distance between the two drill holes, measured with a divider after downfracture, served as the reference for the final vertical maxillary position. This technique relies on a stable, non-displaced reference point on the piriform rim throughout the procedure. In the PSI group, the intermediate splint was inserted only temporarily to identify posterior bony interferences and was not used for definitive maxillary positioning. Mandibular osteosynthesis was performed using the final splint, and fixation of the mandible was achieved with an H-shaped plate (REF 25–394-34–09, KLS Martin SE & Co. KG, KLS Martin Platz 1, 78,532 Tuttlingen, Germany).

Postoperatively, the final splint remained in place for one week, and light orthodontic elastic bands were applied for four weeks in both groups. No postoperative maxillomandibular fixation was performed postoperatively. Postoperative CBCT was acquired two to three days after surgery.

### Data sources and measurement

**Fig. 4:**
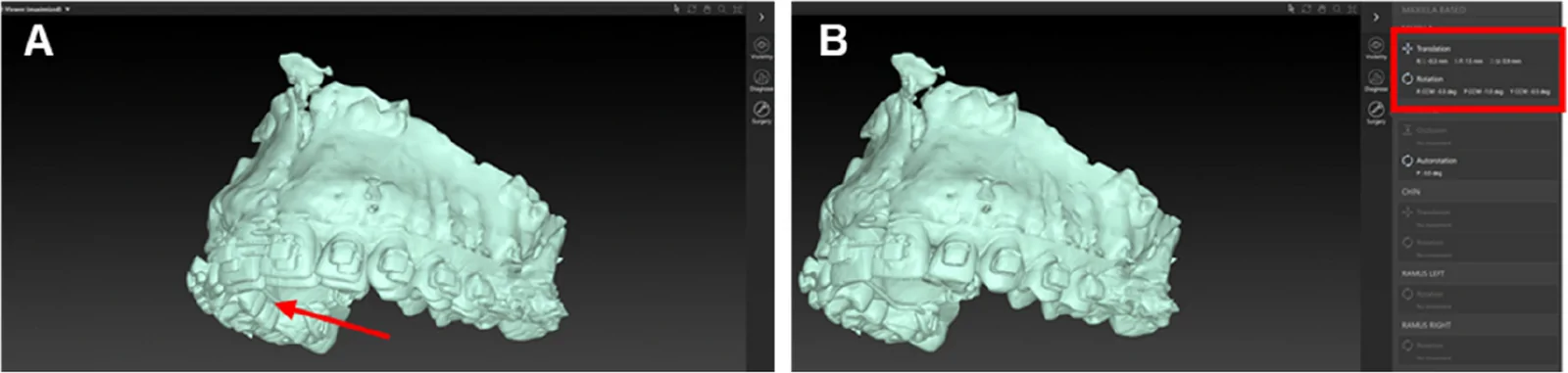
Presents the procedure for data collection within the Compare Models module of the IPS® Case Designer. **A** After matching the non-displaced facial skull, both the planned and postoperative maxillary displacement scenarios are displayed. **B** Following accurate superimposition of the maxillae, the deviation between the planned and achieved postoperative maxillary displacement is provided in the box highlighted in red in the image

The patients’ clinical files served as the source for the demographic data and the Angle classification. For the primary and secondary outcome variables, the IPS® Case Designer (Version 2.7.5.1, KLS Martin, Tuttlingen, Germany) was utilised. The "Compare Models" module was employed to quantify deviations between the postoperative and planned maxillary positions (Fig. 4). Postoperative DICOM datasets, acquired using the same device as the preoperative scan, were imported, and an STL surface of the bone structures was generated. Consistent with preoperative planning, the unchanged preoperative intraoral scans were matched to the postoperative STL surface. Points on bone structures that remained constant throughout surgery were used to align the preoperative and postoperative STL surfaces, thereby visualizing structures that underwent positional changes. Following separation of the upper and lower jaws, the preoperative and postoperative maxillary positions were superimposed to quantify translational and rotational discrepancies (Fig. 4).

The software generated signed values based on the direction of change: rightward shifts were negative, leftward positive; frontal displacement positive, backward negative; upward positive, downward negative; clockwise roll positive, counterclockwise negative when viewed frontally; clockwise pitch positive, counterclockwise negative when viewed from the right; yaw was signed analogously when viewed from below. Absolute values were used for all calculations to facilitate robust comparisons of deviations.

Operating time was defined as the interval between the initial incision and completion of the surgical suture, as documented in the surgical report. The reduced sample size in some operating-time comparisons reflects cases involving maxilla-only surgery or additional genioplasty.

### Bias

Imprecise measurements of translational and rotational deviations from the planned position were addressed by evaluating intraobserver and interobserver variability were evaluated using a two-way random-effects ANOVA model. This approach determined consistency for a single rater and across repeated measurements. Three independent investigators with varying levels of clinical experience each conducted 10 repeated measurements on a randomly selected patient.

Except for the intervention involving maxillary positioning, the treatment protocol was identical for both groups.

A single individual conducted both the planning and final data acquisition for all cases included in the final analysis.

### Sample size

Given the retrospective study design, no a priori sample size calculation was performed. Instead, all consecutive patients who met the predefined eligibility criteria during the 3.5-year study period (January 2020 to June 2023) were included. The sample size was therefore determined by the number of eligible cases available in the institutional database within this predefined period, resulting in a final cohort of 26 patients (11 in the PSI group and 15 in the control group). Given the limited sample size and non-randomised study design, the study was considered exploratory, with the aim of estimating between-group differences and generating hypotheses for subsequent adequately powered prospective studies.

### Statistical analysis

Statistical analyses were performed using Prism 8 (GraphPad Software, Boston, USA). Data distribution was assessed using the Shapiro–Wilk test. Continuous variables are presented as mean ± standard deviation (SD) unless otherwise stated. Between-group comparisons were conducted using unpaired two-tailed *t*-tests for normally distributed data. A two-way analysis of variance (ANOVA) with Fisher's least significant difference (LSD) post hoc test was used to analyse differences across individual translational and rotational movement components, and Tukey's comparison test was used to supplement this. Pearson's correlation coefficient was used to assess associations between planned displacement magnitude and postoperative deviation. Effect sizes (Cohen's *d*) were calculated for primary comparisons to quantify group differences. To complement *p*-values and improve interpretability, 95% confidence intervals (CIs) for effect sizes were computed, providing an estimate of precision and clinical relevance. Instead of relying on post-hoc power calculations, which may be misleading, the results are interpreted based on effect sizes and their associated confidence intervals, in line with current statistical recommendations. Statistical significance was defined as *p* ≤ 0.05. The asterisks defined the level of statistical significance as follows: *p ≤ 0.05; **p ≤ 0.01; ***p ≤ 0.001.

## Results

### Patient characteristics

The study included 26 patients. Of these, 11 patients (five female, six male, aged 18–41 years, 28.4 ± 7.7, 95% CI 23.2–33.5; four Angle Class II and seven Angle Class III) were assigned to the PSI group, while 15 patients (eleven female, four male, aged 17–39 years, 27.4 ± 6.8, 95% CI 23.6–31.2; four Angle Class II and eleven Angle Class III) comprised the control group. Fisher’s exact test indicated no significant differences in gender (*p* = 0.2280) or Angle classification (*p* = 0.6828) between groups. There was no significant difference in age between groups (unpaired *t*-test, *t*(24) = 0.3504; *p* = 0.7291; Cohen's *d* = 0.1391; 95% CI − 4.890 to 6.890).

### Translational and rotational deviation from the planned position

**Fig. 5:**
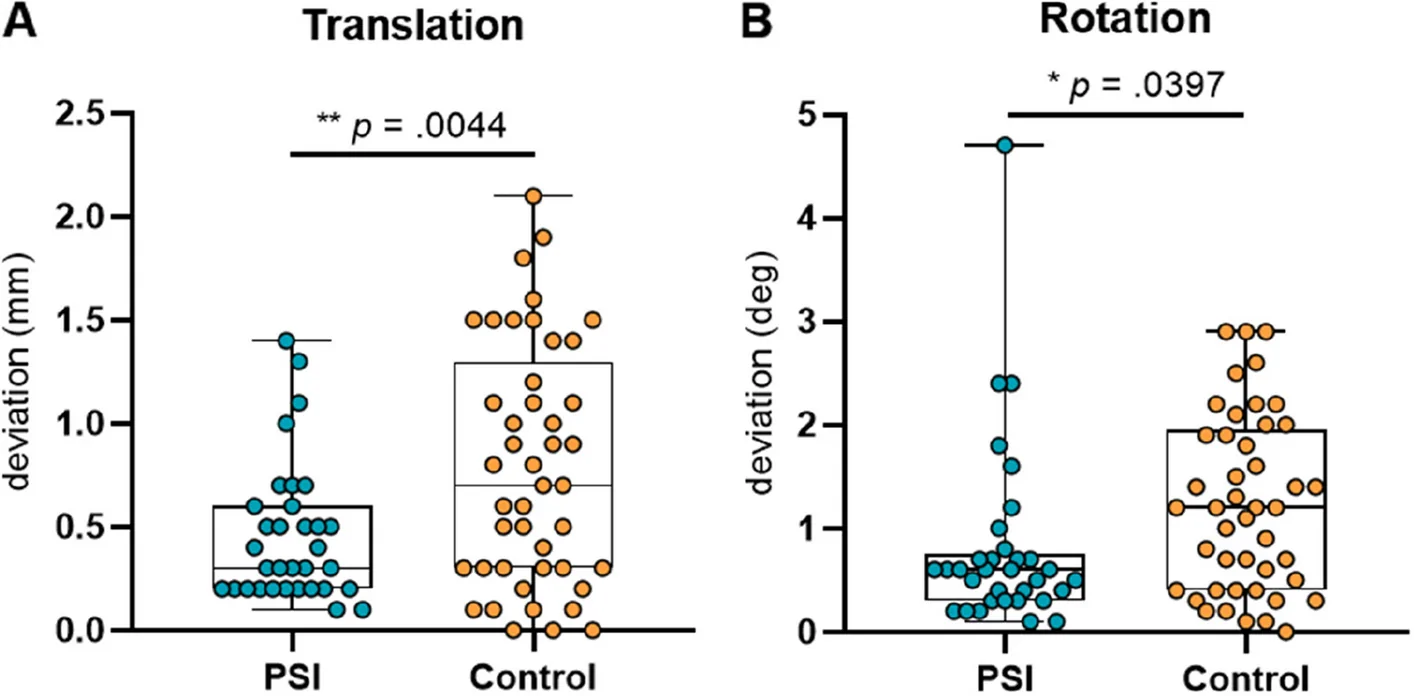
Demonstrates smaller deviation in maxillary placement planning, both in translation and rotation, when PSI are used compared to splint-based positioning. The box plots display discrepancies between planned and actual postoperative maxillary positions for patients treated with PSI-based or splint-based methods. Each box plot includes the median, quartiles, and standard deviations, with individual data points shown. **A** Translational movements. **B** Rotational movements

The mean translational deviation from the planned maxillary position was 0.46 ± 0.33 mm in the PSI group (*n* = 11 × 3 parameters) and 0.79 ± 0.58 mm in the control group (*n* = 15 × 3 parameters). The difference between groups was statistically significant (unpaired *t*-test, *t*(76) = 2.9354; *p* = 0.0044; Cohen's *d* = − 0.6728; 95% CI − 0.5539 to − 0.1061) (Fig. 5A). The mean rotational deviation was 0.82 ± 0.91 degrees in the PSI group and 1.24 ± 0.85 degrees in the control group (unpaired *t*-test, *t*(76) = 2.0926; *p* = 0.0397; Cohen's *d* = − 0.4796; 95% CI − 0.8198 to − 0.0202) (Fig. 5B).

### Deviation by individual movement component

**Fig. 6:**
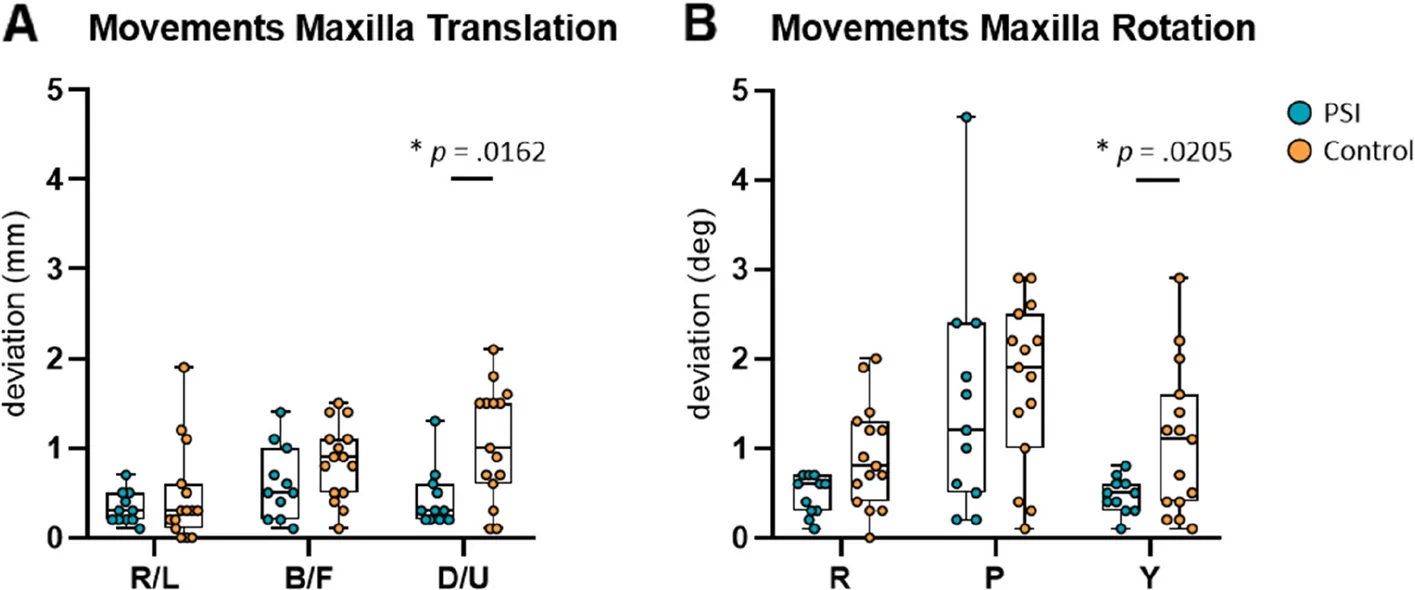
Presents deviations in individual translational and rotational movements. Significant discrepancies are observed in the down/up and yaw movements. The box plots illustrate the extent of discrepancies between the planned maxillary movement and the final position achieved in translational (**A**) and rotational (**B**) movements. Box plots display the median, quartiles, and standard deviations, with individual values shown as points. The data were analysed using a two-way ANOVA with an uncorrected Fisher’s LSD multiple comparison test. PSI: *n* = 11; Control: *n* = 15; D/U: *p = 0.0162; Y: *p = 0.0205; Movements: R/L = right/left; B/F = back/front; D/U = down/up; R = roll; P = pitch; Y = yaw

A two-way ANOVA with an uncorrected Fisher’s LSD multiple comparison test identified statistically significant between-group differences in the down/up translation (*p* = 0.0162) and yaw rotation (*p* = 0.0205) components. The mean down/up deviation was 0.44 ± 0.34 mm for the PSI group (*n* = 11 parameters) and 1.06 ± 0.63 mm for the control group (*n* = 15 parameters) and showed significant difference (unpaired *t*-test, *t*(24) = 2.9533; *p* = 0.0069; Cohen's *d* = − 1.1723; 95% CI − 1.0533 to − 0.1867). The mean yaw deviation was 0.47 ± 0.20 degrees for the PSI group (*n* = 11 parameters) and 1.07 ± 0.83 degrees for the control group (*n* = 15 parameters), which differed significantly between groups (unpaired *t*-test, *t*(24) = 2.3364; *p* = 0.0282; Cohen's *d* = − 0.9275; 95% CI − 1.1300 to − 0.0700). No statistically significant between-group differences were found for the remaining translational (right/left, back/front) or rotational (roll, pitch) components. Pitch deviation was numerically the largest among all rotational components in both groups (PSI: 1.51 ± 1.32 degrees, *n* = 11; control: 1.72 ± 0.92 degrees, *n* = 15). Individual translational and rotational movement components are presented in Fig. 6.

### Association between planned displacement magnitude and deviation

**Fig. 7:**
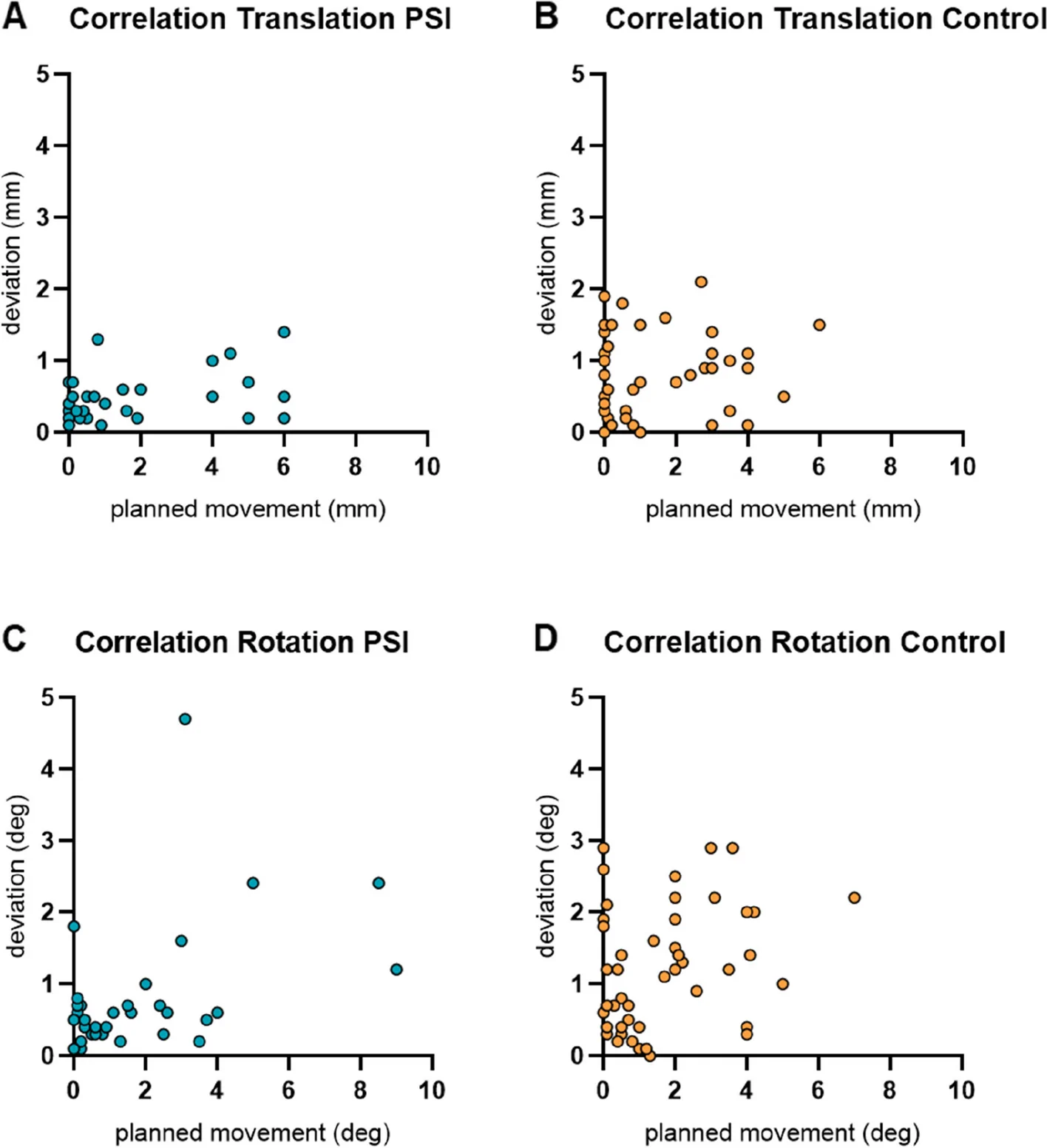
Demonstrates a positive average correlation between the deviation from the planned maxillary displacement and both the magnitude and angle of displacement in the PSI group, as measured by the Pearson correlation coefficient (**A**) Correlation translation PSI group: *r* = 0.42 (*p = 0.0162; *n* = 11 × 6 parameters); (**B**) Correlation translation control group: *r* = 0.096 (*p* = 0.5296; *n* = 15 × 6 parameters); (**C**) Correlation rotation PSI group: *r* = 0.45 (**p = 0.0088; *n* = 11 × 6 parameters); (**D**) Correlation rotation control group: *r* = 0.27 (*p* = 0.0963; *n* = 15 × 6 parameters)

In the PSI group, deviation from the planned position showed a significant correlation with the magnitude of planned displacement for both translation (*r* = 0.42, *p* = 0.0162; Fig. 7A) and rotation (*r* = 0.45, *p* = 0.0088; Fig. 7C). In contrast, the control group demonstrated no significant correlation for translation (*r* = 0.10, *p* = 0.5296; Fig. 7B) or rotation (*r* = 0.27, *p* = 0.0693; Fig. 7D). Additionally, neither group exhibited a correlation when individual movement components were analysed separately.

### Operating time

**Fig. 8:**
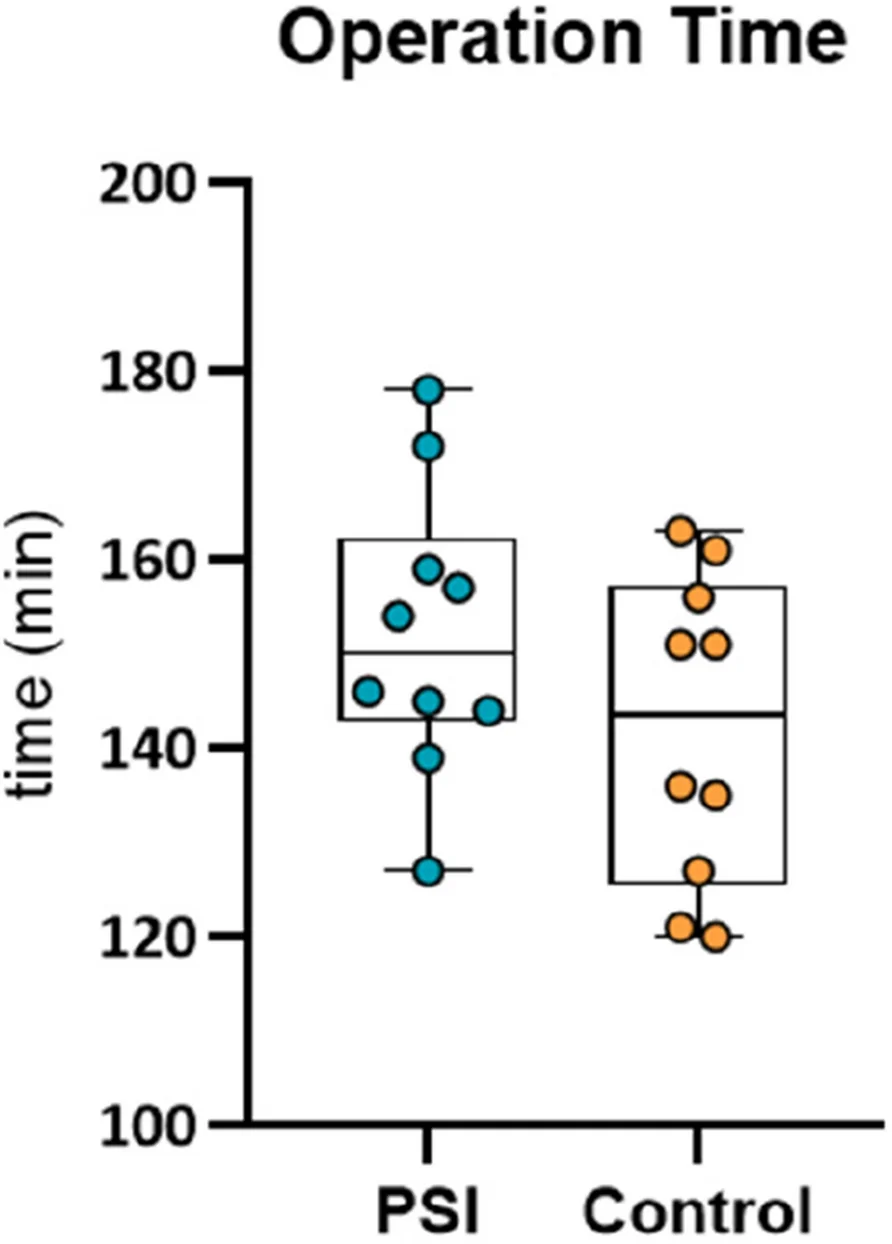
Presents the duration of surgical procedures of both groups. The box plot illustrates the median, quartiles, and standard deviations, with individual data points displayed

The mean operating time (Fig. 8) was 152.1 ± 15.26 min in the PSI group (*n* = 10) and 142.1 ± 16.31 min in the control group (*n* = 10). This difference was not statistically different (unpaired *t*-test, *t*(18) = 1.416; *p* = 0.1739; Cohen's *d* = 0.6332; 95% CI − 4.8392 to 24.8392).

### Reproducibility

Intraclass correlation coefficients (ICC) were as follows: 0.80 for right/left translation, 0.83 for back/front translation, 0.79 for down/up translation, 0.55 for roll, 0.70 for pitch, and 0.88 for yaw. These values indicate moderate (0.5–0.75) and good (0.75–0.9) reliability.

## Discussion

### Key findings

The deviation from the planned maxillary position was smaller in the PSI group compared to the control group for both translational and rotational movements, with this difference reaching statistical significance. Analysis of individual movement components revealed that only the down/up translation and yaw rotation components differed significantly between groups. In the PSI group, deviation increased with the magnitude of planned displacement, whereas this trend was not observed in the control group. Operating time did not differ significantly between groups. No statistically significant baseline differences in age, gender, or Angle classification were observed between groups. However, due to the retrospective, non-randomised design, this finding indicates limited baseline comparability across the measured variables rather than true homogeneity. Additionally, unmeasured differences in case complexity or surgeon preference cannot be excluded.

### Strengths and limitations

This study directly compared translational and rotational deviations using a validated software-based superimposition method, assessed all six axes of movement rather than translation alone, and formally evaluated both intra- and interobserver reliability. Both groups were treated at the same institution, by the same surgical team, and within a defined time period, thereby limiting variability attributable to operator or setting.

Several limitations should be acknowledged. First, the retrospective non-randomised design precludes causal inference. Second, allocation was determined by the logistical feasibility of PSI manufacturing, which may have introduced selection bias. Third, the small sample size limited the ability to adjust for potential confounders, including asymmetry severity and concomitant procedures. Fourth, the evaluated protocol constituted a hybrid rather than a fully splintless PSI workflow. Finally, data on patient-reported outcomes, long-term skeletal stability, and comprehensive cost-effectiveness analyses were not available.

### Interpretation

The accurate transfer of the virtual surgical plan to the operating theatre remains a principal challenge in orthognathic surgery. Conventional maxillary positioning relies on intermediate splints and rotational positioning of the maxillomandibular complex within the temporomandibular joints, which makes the transfer process susceptible to cumulative errors during condylar seating, manual positioning, and fixation. PSIs have been introduced to mitigate these sources of transfer error by enabling direct translation of the virtual plan to the surgical field [19, 20].

Although PSI-assisted surgery has gained popularity, the existing literature is heterogeneous regarding study design, patient selection, outcome assessment, and definitions of splintless surgery. As a result, both the extent of any accuracy advantage and its clinical relevance remain unclear. Recent systematic reviews indicate that PSI workflows typically enhance geometric accuracy. However, the certainty of this evidence is low to moderate, and the clinical significance of these improvements has not been established [20–22].

Reported translational deviations in patient-specific instrument PSI-assisted workflows display considerable variability, with minimal inaccuracy as low as 0.03 mm and a median maximum inaccuracy of 0.1 mm when non-absolute values are used. This method may substantially underestimate true deviation, as positive and negative errors can offset each other [13]. The mean translational deviation has been reported as 0.88 mm for PSI, compared to 1.04 mm for splint-based positioning [14]. Multiple studies, consistent with the present findings, demonstrate a statistically significant advantage for PSI [16, 23]. In the current cohort, mean translational deviation ranged from 0.45 to 0.79 mm in the PSI group and from 0.8 to 1.26 mm in the control group. Both groups remained below the approximately 2 mm threshold generally considered clinically acceptable for maxillary positioning [24–26]. Reported rotational deviation with PSIs ranges from 0.43 to 2.44 degrees [14–16], which is consistent with the values observed in this study.

A likely explanation for the observed between-group difference in down/up translation is that vertical positioning in the PSI group was determined by the pre-drilled pilot holes of the cutting guide. In contrast, the control group depended on intraoperative paranasal reference drillings, a method prone to error when rotational movement or substantial anteroposterior displacement occurs. Alternatives, including incisal-point measurement [27] and intraoperative navigation [28], have been suggested to mitigate these errors. Although some studies have reported improved anteroposterior movement accuracy [12, 16], this improvement was not observed in the present analysis, whereas improvements in other directions were generally consistent. Pitch deviation constituted the largest rotational component in both groups. Because osteosynthesis is limited to the anterior maxilla, incomplete removal of posterior bony interferences during downfracture may permit the maxilla to shift distally toward its original position due to subsequent mandibular manipulation. This mechanism, also described by Li et al. [15], aligns with reports that planned counterclockwise rotation can result in unintended distal-caudal displacement [15, 29]. While a clear osteotomy line can be established anteriorly, a random fracture line typically forms posteriorly during downfracture, creating interfering contacts that, if not eliminated, may cause pitch and other positional errors [15]. Identifying and removing these interferences without a splint is difficult. In splint-based surgery, this is accomplished through autorotation at the temporomandibular joint with the maxilla fixed to the mandible, a reference point not available in a purely splintless PSI protocol, especially for clockwise rotations or cranial impaction. This challenge, also noted by Kraeima et al. [12] and Li et al. [17], supports the rationale for the temporary intermediate splint step used in the PSI-based hybrid protocol examined in this study. The splint served solely to identify and remove posterior bony interferences, not for final positioning or fixation. The significant yaw finding did not persist in the more conservative Tukey comparison, consistent with Kraeima et al. [12], and a false-positive result cannot be excluded given the high standard deviation of the yaw data.

The observed weak correlation between planned displacement magnitude and postoperative deviation in the PSI group aligns with the findings of Imai et al. [30], although that study did not assess rotational parameters. Brunso et al. reported increased deviation at planned displacements of 10 mm or greater [31], while Kraeima et al. found that PSI performed better for anteroposterior translations of 3.67 mm or larger [12]. In contrast, no comparable fixed threshold was identified in the present dataset, and no correlation was observed when individual movement components were analysed separately. Consequently, this association should be interpreted with caution.

Although several previous studies have reported reduced operating times with PSI use [5, 11, 13, 16], the present study did not observe a reduction. Instead, operating time was numerically longer in the PSI group. This outcome may result from the additional intermediate-splint step required to address distal bony interferences. Additionally, preoperative planning, manufacturing lead time, and sterilisation logistics for the PSI, which were not included in this dataset, constitute further time costs not reflected in intraoperative duration. Rückschloß et al. also identified increased cost, delivery time, and intraoperative changes as key disadvantages of PSIs [16].

Statistically significant improvements in geometric accuracy should not be assumed to translate into clinically meaningful benefits. Systematic reviews indicate that while patient-specific implant (PSI)-assisted workflows reduce deviations from the virtual surgical plan, there is limited evidence for improvements in functional outcomes, occlusion, patient satisfaction, or long-term skeletal stability [20, 21, 32]. In this study, study, the observed improvements of approximately 0.3 mm and 0.5° are unlikely to result in measurable clinical differences for most routine orthognathic procedures. These values fall below the threshold of clinical perceptibility and are substantially less than the approximately 2 mm threshold generally considered clinically relevant in this context [24–26]. The 95% confidence intervals for the effect sizes were wide and included the possibility of no effect for both comparisons, further limiting confidence in the clinical significance of these findings. No reduction in operating time was observed. The evaluated workflow required temporary use of an intermediate splint to remove bony interferences before definitive splintless fixation, resulting in a hybrid rather than a fully splintless protocol. The adjunctive splint step may have contributed to the observed accuracy, and its relative impact compared to the PSI itself cannot be determined from this dataset.

Overall, these findings do not support the routine adoption of PSI-assisted maxillary positioning over conventional splint-based techniques based solely on accuracy. Selective application in complex cases, such as those involving pronounced facial asymmetry or syndromic anatomy where precise rotational control is critical, may be more appropriate pending additional evidence.

This study demonstrates that a hybrid PSI-based hybrid workflow can reduce geometric deviations from the virtual surgical plan compared to conventional splint-based positioning. However, the improvements observed were modest and should be interpreted in the context of a retrospective, non-randomised study that evaluated a hybrid rather than a fully splintless workflow. Future prospective randomised studies are necessary to determine whether these gains in geometric accuracy result in clinically meaningful improvements in patient outcomes and cost-effectiveness.

## Conclusion

In this retrospective cohort, PSI-based hybrid maxillary positioning resulted in statistically smaller deviations from the virtual surgical plan compared to conventional splint-based positioning. Despite these findings, the absolute differences were minimal, there was no observed reduction in operating time, and the PSI workflow necessitated temporary use of an intermediate splint to address bony interferences prior to definitive splintless fixation. Therefore, the clinical significance of the observed improvement in accuracy is limited. Larger prospective randomised studies assessing fully splintless PSI workflows, patient-centred outcomes, and comprehensive cost-effectiveness analyses are necessary before routine implementation can be recommended.

## Data Availability

The datasets generated during and analysed during the current study are available from the corresponding author upon reasonable request.

## Abbreviations

ANOVA: Analysis of variance
B/F: Back/front
CAD: Computer-aided design
CAM: Computer-aided manufacturing
CBCT: Cone beam computed tomography
D/U: Down/up
LSD: Least significant difference
ICC: Intraclass correlation coefficient
IPS: Individual patient solutions
min: Minutes
MMF: Maxillomandibular fixation
n: Number
P: Pitch
PSI: Patient-specific implants (PSIs)
R: Roll
R/L: Right/left
STL: Standard tessellation language
Y: Yaw
